# Perspectives on post-violence support-seeking and service provision among adolescent girls and young women: Insights from community conversations in Lusaka, Zambia

**DOI:** 10.1177/17455057261435814

**Published:** 2026-04-03

**Authors:** Christina Laurenzi, Chuma Busakhwe, Chanda Mwamba, Chipo Mutambo, Eugene Mupakile, Elona Toska

**Affiliations:** 1 Institute for Life Course Health Research, Department of Global Health, Stellenbosch University, Tygerberg, South Africa; 2 Accelerate Research Hub, Centre for Social Science Research, University of Cape Town, South Africa; 3 Centre for Infectious Disease Research Zambia, Lusaka, Zambia; 4 Paediatric-Adolescent Treatment Africa, Mowbray, Cape Town, South Africa; 5 Kabangwe Creative Initiative Association, Kabangwe, Zambia; 6 Department of Social Policy and Intervention, University of Oxford, UK

**Keywords:** gender norms, community conversations, adolescent girls and young women, help-seeking, post-violence service provision

## Abstract

**Background:**

Adolescence is a pivotal time for growth and autonomy, with gender norms influencing how individuals experience this developmental transition. These norms become more complex in the context of gender-based violence (GBV), which is common across adolescent girls globally. Alongside societal and gender norms, systems governing health services, social care, and governmental resource allocation also significantly shape responses to GBV. To move toward healthier norms, it is critical to better disentangle how gender norms operate, especially in the context of experiences of violence, and how these norms shape post-violence support-seeking and service provision.

**Objectives:**

We set out to explore the factors that shape support- and service-seeking, and service provision, for adolescent girls and young women who experience violence.

**Design:**

An exploratory qualitative design was used for the research.

**Methods:**

We conducted a series of community conversations in peri-urban Lusaka, Zambia in October 2022. Purposively recruited participants included adolescent girls and young women aged 15–24 (*n* = 12), healthcare professionals (*n* = 10), and community members (*n* = 8). Community conversations combined arts-based and participatory methods with group discussions, and were conducted by an experienced Zambian female researcher over 5 days. All necessary ethical approvals were obtained and key procedures followed.

**Results:**

Participants spoke about different motivations and drivers to seek support for GBV, revealing multiple layers of support-seeking, as well as stigmatization. Emerging narratives also spoke to the norms, practices, and resources that shape service provision. In sharing personal anecdotes and perceptions of available post-violence support, participants tapped into deeper underlying norms and expectations surrounding how violence against girls and women is managed—silence, secrecy, and acceptance among them—as well as actual narratives on service provision.

**Conclusion:**

Our study’s insights, while context-specific, also speak to broader considerations for norms shifting. Harnessing safer norms across generational and sectoral divides may reduce violence and improve service access among adolescent girls and young women.

## Introduction

Adolescence and young adulthood are periods of change, characterized by layered social transitions alongside identity development and a growing sense of self. As adolescents navigate multiple decisions and societal expectations at a pivotal stage of life, gender norms can deeply shape their social interactions and affect how they engage with social systems and services.^1^ Gender norms, according to Cislaghi and Heise, suggest acceptable and appropriate actions for women and men in a given group. Because gender norms are “embedded in formal and informal institutions, nested in the mind, and produced and reproduced through social interaction,” these authors argued that gender norms “play a role in shaping women and men’s (often unequal) access to resources and freedoms, thus affecting their voice, power and sense of self.”^2^

While these effects can be protective at times and support individual growth or autonomy, there is also clear evidence of how inequitable gender norms can lead to long-term harm. Several recent studies, including the Global Early Adolescent Study, have focused on how gender and power inequalities in early adolescence shape health and development outcomes,^3,4^ including later into adulthood.^5^ Our previous work has identified powerful effects of health provider stigma, as well as more pervasive social scripts around violence, that deter adolescent girls from seeking necessary healthcare.^6–9^

These norms become more complex in the context of gender-based violence (GBV). Recent estimates indicate that one in four ever-partnered adolescent girls aged 15–19 years have experienced intimate partner violence globally.^10^ GBV is perpetuated by inequitable gender norms and social structures, and gender and social norms play a critical role in shaping how individuals, communities, and systems respond to these incidents.^11,12^ Commonly held beliefs or norms about what counts as violence can influence whether a survivor seeks support in the aftermath of experiencing GBV, and from who and where.^13^ These decisions may also be driven by the structure of social networks and relationship dynamics between adolescent girls and their peers, partners, and families.

However, as Cislaghi and Heise noted in their exploration of common pitfalls in social norms interventions, the “matrix of factors” that sustain harmful practices means that social norms cannot be isolated as the sole drivers of GBV and are part of more complex systems.^14^ Alongside societal and gender norms, systems governing health services, social care, and governmental resource allocation also play a significant role in shaping responses to GBV. Service delivery systems, and the providers who work within them, may be insufficiently attuned to the unique needs of survivors, failing to consider their experiences of violence.^12^ For adolescents, who are sensitive and cognitively respond positively to responsive, non-judgmental care, negative interactions in the aftermath of GBV may connote blame or shame, rather than helpfulness.^15^ Another challenge is that across multiple levels of service delivery, there are limited resources allocated to GBV prevention and response, which often hampers the effectiveness of interventions.^16^ These factors can further exacerbate the challenges that survivors face when seeking support, making it even more difficult for them to access the care and resources they need.

There is a growing recognition that more sustained embedded efforts are needed to shift harmful gender norms and promote healthier norms within specific contexts.^17^ Healthier norms, in turn, can promote better physical health, mental well-being, and human flourishing. Shifting harmful norms is closely linked with public health promotion, as norms have the power to influence tangible actions that ultimately shape health outcomes.^18^ However, in order to move toward healthier norms, it is critical to better disentangle how gender norms operate, especially in the context of experiences of violence, and how these norms shape post-violence support-seeking and service provision.

This work needs to recognize both the universal, common drivers of violence alongside the specific contexts and social relationships that perpetuate GBV in a given place. To contextualize the role of norms and common drivers of violence in post-violence care, our team conducted a series of community conversations with young women, community leaders and members, and community-based service providers, in one community in peri-urban Lusaka, Zambia (peri-urban indicating on the outskirts of the city center). In earlier programmatic work focused on providing peer-delivered mental health support for adolescent girls living with HIV^6^ our academic–non-governmental organization (NGO) partnership identified GBV as a recurring issue. These community conversations provided a context for better understanding drivers of GBV and areas for intervention. They also served as the first activity embedded in a longer-term intervention study to equip peer supporters with better skills for screening and referral, called Screen & Support. An earlier analysis of these conversations, specifically examining data from three groups of adolescent girls and young women, identified how “social scripts” of violence can play a central role in how violence is understood and tolerated.^6^ It also focused on how norms can take on new significance, and become activated, when young women experience violence.

This analysis aims to extend prior analysis to consider how these norms influence actions taken in the aftermath of violence—and how they interact with existing resources and service models. Specifically, this article explores the factors that shape support- and service-seeking, and service provision, for adolescent girls and young women who experience violence.

## Methods

This study was conducted between May 2022 and April 2023, with the primary research reported on in this manuscript collected in October 2022, in a peri-urban area on the outskirts of Lusaka, Zambia, where young women face high rates of GBV. The study site was selected based on the research team’s existing relationships with two organizations: Pediatric-Adolescent Treatment Africa (PATA) and Kabangwe Creative Initiative Association (KCIA). Both organizations have extensive experience working with adolescents living with HIV.

Our partners at KCIA supported the team in identifying and purposively recruiting a total of 30 individuals through community-based networks to participate in formative community conversations. Participants included three core groups. The first was adolescent girls and young women aged 15–24 (*n* = 12); adolescent girls and young women were recruited according to diverse criteria to represent distinct profiles: survivors of GBV, those living with HIV, and young women who were married. Healthcare professionals (*n* = 10) included nurses, peer supporters, and social workers embedded in healthcare spaces. The final group, community members (*n* = 8), included teachers, religious leaders, local political leadership, and a representative from the local market association. Regarding inclusion criteria, eligible participants met the above core “profile” criteria and the age range; any participants who were unable to provide consent or assent for themselves were excluded. We aimed for small groups to encourage dynamic engagement and exchange, with purposeful inclusion of key constituent groups; for adolescent girls and young women, we specifically looked to keep groups small for purposes of safety, comfort, and confidentiality. All participants provided written informed consent, and adolescent girls under 18 years provided written assent alongside parental or guardian written consent. The consent procedures included information about the study, participants’ involvement, and available referral options due to the sensitive nature of the session content.

Data was collected using community conversations methods, which combined arts-based and participatory methods (such as journey maps, personas, and free-listing) with group discussions.^19–23^ An experienced Zambian female researcher with a master’s degree and extensive qualitative research experience and training (CM) led these engagements in a central neutral location in the participants’ community; sessions lasted between 3 and 4 h, on average and were observed by two additional research team members and one community partner. Separate sessions, spanning 4 days in October 2022, were convened for GBV survivors (*n* = 4), adolescent girls and young women living with HIV (*n* = 4), young married women (*n* = 4), healthcare professionals (*n* = 10), and community members (*n* = 8). A final session brought together a cross-section of prior participants. We included different stakeholders to capture diverse lived experiences and GBV contexts, gather information on available GBV support resources, and gain insights that could inform the practical aspects of implementing the screening and support package.

Additionally, discussion guides were tailored for each group to facilitate more focused, in-depth conversations (see Supplemental Material). Core topics, explored with each group, enabled rich data to be collected while inviting diverse perspectives. The discussions were mainly conducted in a combination of local languages (Nyanja and Bemba), with some participants using English. All sessions were audio-recorded and transcribed and translated into English for analysis; additional arts-based products and lists were photographed, catalogued, and translated as needed prior to analysis. At the end of these formative community conversations, preliminary debriefing and data review was conducted and the research team was satisfied that data saturation had been met.

Data were thematically analyzed using Dedoose, a web-based software platform for analyzing qualitative and mixed methods research data.^24^ In the initial coding round, broad codes were developed from one group’s full-day transcript by two coders. These high-level codes were reviewed and refined using a second transcript, resulting in a final coding framework, which was then applied to all group transcripts evenly and cross-checked against arts-based products, where these were described on the audio recordings. The codes were compared, and themes and sub-themes were discussed with the larger study and authorship team, as well as with expert consultants, before being finalized and written up. Arts-based products were used to triangulate findings as well as provide further context to recorded conversations.

The research team, comprised of South Africa- and Zambia-based researchers and implementing partners, engaged in several practices to ensure reflexivity and reduce risk of bias. Multiple strategies were used to enhance trustworthiness, including mechanisms to enhance credibility, dependability, and confirmability. The research team engaged with participants over multiple hours, in a combination of informal and formal activities designed to build rapport and comfort, including sharing a meal. Our research processes were carefully documented, with a diverse, experienced team engaged at different stages to ensure the rigor of the data analysis and interpretation of findings. Team debriefs after each session and during the data analysis process was paired with collaborative discussions and interpretation of findings with support from our local NGO partner to ensure accuracy and minimize researcher bias; due to logistical constraints, we did not engage with all participants for member-checking.

Ethical approval was granted by Centre for Social Science Research, University of Cape Town (CSSR 2022/03), ERES Converge Ethics Board, Lusaka, Zambia (19 August 2022), and the National Health Research Authority, Zambia (NHRA-313/05/10/2022). The reporting of this study conforms to the COREQ statement.^25^

## Results

We organized our findings into three core thematic areas: factors shaping support-seeking, factors shaping service provision, and shifts in norms and practices (Table 1).

**Table 1.** Identified themes.

Themes	Subthemes
Factors shaping support-seeking	Normative views on relationship expectations and normalization of violence
	Knowledge and perceptions of available services and resources
	Navigating individual motivations, family considerations, and support systems
Factors shaping service provision	Norms pervading service provision
	Complexity in the post-violence services cascade
	Resource limitations compounding existing challenges in seeking justice
Shifts in norms and practices	

### Factors shaping support-seeking

Participants spoke about different motivations and drivers to seek support for GBV, revealing multiple layers of support-seeking. This included social support that was often sought from family and more “traditional” spaces that were based around religion and community, as well as more formalized pathways for support-seeking that include healthcare services, religious counsel, and police/legal services. Sometimes women themselves felt able to make these choices; other times, especially for younger women, these considerations were shaped by their families.

#### Normative views on relationship expectations and normalization of violence

Participants across all groups spoke about the power of normative views on marriage, womanhood, and violence. They acknowledged deeply held beliefs about women’s responsibilities in relationships to endure violence and evoked narratives of violence being intergenerationally passed on, and justified. These norms shaped the willingness of women, especially young women, to seek support after experiencing violence.

Participant 4: “Families also put women [under] pressure of staying in marriages”

Participant 1: “The pressure to stay is there, the pressure is there. . .”

Participant 3: “They even say when you leave, where will you stay? or keep the children?” (Adolescents/young women)

“There was one family in some area I was staying, he used to beat the wife and the children. Why do you beat your children? Because they also used to beat me. They told me that is what you should do, when you get married, you must beat! You see, so it affects. . .someone’s growth, the growing. You tend to bring the same situation you were in and take it as the correct thing that your supposed to do.” (Healthcare worker)

Stigma and judgment from family members was specifically noted, contributing to a sense of helplessness and limiting options for exiting marriages. In these instances, norms might shape how willing family members may be to help; to let the matter dissipate, so as to not cause further collateral damage in the community; or to actively discourage or dissuade young women from acting. Additionally, judgment and lack of support from family members created a strong pressure to remain within the marriage, even when it was harmful or unhealthy.

“Then stigma from family members, these families we come from are difficult. They are even the ones who will say, ‘don’t say anything, who will buy us food at home? If you tell, who will be buying us food at home?’ So just stay in that marriage, even if you are being beaten but they will say just stay, and you will stay suffering.” (Healthcare worker)

“Another reason is lack of support from family members. You find she will leave and go and tell them, but they will tell her, you have also gone through this. You have to be strong in marriage, go back. So, they fear to tell someone because they will be told they are wrong.” (Community member)

Emerging from these conversations were also narratives of women withstanding violence to avoid marriages ending, framing the complexity of ongoing marital conflict. Despite experiencing marital dissatisfaction, some individuals remained within their marriages because of traditional beliefs that view divorce or separation as failure.

“Some hide, they will be beaten, but the husband says if you tell anyone I will divorce you, and she still loves the husband. If you tell anyone that I have beaten [you], you the marriage will end. In the morning, instead of saying the truth, she will say my head hurts. . .That is why they hide.” (Community member)

#### Knowledge and perceptions of available services and resources

Participants also spoke about young women having limited knowledge of what constitutes GBV, and emphasized the role of sensitization in supporting young women to recognize signs of violence and seek support. One healthcare worker shared how an awareness session translated into a young women understanding her situation and deciding to seek support:

“We had a lot of sessions in [two peri-urban areas], so she said it’s useless, she told us so herself, they are just talking, but once we talked about the types of GBV, she became alert. So, what he does, insulting me, when he walks in, ‘you daughter of a dog,’ this and that. She was being abused verbally, after she knew she went to her parents who helped her. She also went to a friend, right? She helped her, my friend, what he is doing is a crime, the insults, the beatings. So, she was helped, she went through the process, the police, medicals and staff. The husband was called and is now in order, although they are still together.” (Healthcare worker)

In some cases, there were more significant barriers to overcome in empowering young women experiencing violence. Another healthcare worker spoke about a survivor who she felt did not have agency to leave an abusive marriage:

“I remember encountering one young lady whose husband would always lock the house. And he would leave her outside with the child. So, she would have to wait outside the whole day afternoon with no food. And I asked her. . .you need to do something about this, you need to get out. And of course, I told her this is abuse you are going through. But she felt like it’s okay, I. . .am used to this kind of life, and the capacity to make decisions for their own life is limited to some extent.” (Healthcare worker)

Echoing this lack of awareness and empowerment, one young woman who was married spoke about the propensity for social stigmatization and judgment, instead of learning from each other, which she described as ignorance:

“Another thing is ignorance in the community. Instead of sitting down with my friend asking her why she left her marriage, so that maybe I can learn a thing or two, or what has caused her to leave her home is something that I am also experiencing, we just start to laugh. If something is good, people talk; if it’s bad, they still talk. So it’s lack of knowledge.” (Young married woman)

#### Navigating individual motivations, family considerations, and support systems

Participants who had experienced violence, whether in their marriages or by non-partners, described confusion, self-blame, and shame in the moments and days following these events. In reflecting on pathways and prospects for support in the aftermath of violence, young women spoke about a lack of clarity about next steps. They also described the need to weigh up both available and appropriate options, which were not always the same, and to balance individual needs and well-being with considerations about their contexts. For one young survivor, forgiveness and forgetting was both a path to harmony as well as to personal peace:

“After some days I was back to myself, so had to forget everything and to forgive the person who did this to me and I had hope that God will teach him a lesson.” (Young GBV survivor, journey map)

For some young women, their families decided how to proceed, with or without their input. While limited family support and stigma against help-seeking was cited as a barrier to disclosing violence for young women experiencing violence within marriages, violence victimization by strangers was more often seen as socially unacceptable. One participant detailed her own experience, recounting what happened in the aftermath of the assault on her and her sister:

Participant: “I did not know that the man had come with his uncle to table the issue and for them to know if it was him. He entered the house, Mum had gone to her friend’s house to explain what happened. That is how we told them to enter, and they entered, that man and his uncle.”

Facilitator: “The same man who did the same thing [perpetrator] came?”

Participant: “Yes, with his uncle.”

Facilitator: “After how many days?”

Participant: “The same day in the evening, they heard that people had come looking for them.”

Facilitator: “Who went to look for them?”

Participant: “They just came. . .We all sat down, the whole family and we started talking. So when they asked where it happened, he said at my house. Then they said tomorrow we will take you to the clinic and the same man will provide transport. That is how they went and discussed and they left. In the morning, they brought us to [a one-stop centre], we needed money to go to [the district hospital]. The same man did not remove money, it was his uncle who gave my grandmother. That is how went.” (Young GBV survivor)

In this case, the resolution process took priority over other individual concerns. The participant spoke about attending a one-stop center, where multiple services—health, psychosocial, and legal—were available under one roof. In other instances, family members or friends were described as important in providing support to seek help and follow through with pursuing a legal course of action, in line with survivors’ wishes. These instances were described in response to both marital violence and non-partner violence.

“You need someone to support you. You need someone by your side, because alone you won’t manage to go through the process. When you have someone supporting you, it becomes easy. But when you’re alone you feel like everything is the opposite. It can be friends, sisters, your mother. Maybe one doesn’t know where to report the GBV case, she can go to these people to ask for help on where to go.” (Young married woman)

### Factors shaping service provision

Participants also spoke at length about the norms, practices, and resources that shaped service provision. These factors most often impeded the quality and coordination of services able to be provided. As several participants were service providers themselves—involved in providing health, social/community, and legal services for survivors of violence—they offered important perspectives alongside the narratives and experiences shared in separate sessions by adolescent girls and young women.

#### Norms pervading service provision

As participants described services provided in the aftermath of violence, bureaucratic and institutional power dynamics were common. Narratives of being deprioritized, or cases being mishandled, reflected some of the same gender norms as earlier described. Young survivors of violence recalled feeling dismissed and overlooked.

“The help that they [the police] did not give me, they were not serious. When we went to report they looked indifferent, like they did not want, they did not call.” (Young GBV survivor)

One young survivor also relayed how police handled her case, noting that police were communicating with and providing updates to the family of the perpetrator, but not the survivor. Her peer in the focus group reflected on this story:

“They shouldn’t have been talking on the phone. Even as the papers moved at each point, up to when they got lost, they should have called both families and said the papers are lost, what step will we take? Not just saying the papers are lost, so the case is on standstill, because this one [*indicating the survivor*] is also a child. They tried to hush up the case, maybe because the other person had a higher economic status.” (Young GBV survivor)

Another participant noted that there were certain options that were only open for couples who were married—and that single mothers experiencing relationship difficulties were unable to access these services.

Participant: “They are those who, say for instance, if you are married, the *bana chimbusa* [traditional marriage counselors] will sit down with the couple and counsel them, telling them how they should live. But for us who are not married, we just face them [*laughs*], we just face them.”

Facilitator: “Okay so you have a child, but you are not married. How is your situation?”

Participant: “I am a single parent [*laughs*], being a single parent is hard, we go through a lot of things” (Adolescent/young woman)

#### Complexity in the post-violence services cascade

Beyond norms that shaped services and at times dismissed the seriousness of survivors’ stories, participants also described the processes for seeking post-violence care. In spaces dedicated to GBV specifically, some participants described generally positive experiences. One survivor who was physically assaulted by her neighbor spoke about the process of reporting her case at the clinic’s GBV office, while another survivor spoke about receiving specific health services in the wake of a sexual assault:

Participant: “It was an office with people, people who are just okay.”

Facilitator: “So they just asked you questions?”

Participant: “Yes, they asked me questions like they do at the police station, but without intimidation. I gave a statement on the date, the second time they came, just like that.” (Young GBV survivor)

“[I] am grateful for what they did at the clinic when grandma took us. We were given medicine to protect us from HIV/AIDS and they also gave us injections.” (Young GBV survivor)

Others, however, described a process that was fraught with complexity. While participants noted that Zambia had recently made all GBV-related health services free of charge, multiple service points and circuitous pathways to care were described—some of which required fees. One healthcare worker explained a recent patient she had encountered:

“A woman came, she came, she had injuries and was told to go and get a police report. . . She went to the facility and came for suturing and cleaning the wound. So she came with following day with a police report. And she was looking now for the, what’s this, medical report. She was told again to buy, to pay a k150, yes k150 of which she didn’t have, she even insult[ed], ‘this is nonsense, I started coming and you don’t help me, let me go home.’ So [I] am not sure if she completed the process or what.” (Healthcare worker)

These barriers to service access sometimes exposed survivors to secondary victimization or additional scrutiny, even if unintentionally. This theme was echoed in participants’ arts-based narratives, through the fictional persona of Grace, who sought clinical care for physical abuse by her husband:

“At the hospital [Grace] found a doctor and he explained that he could not attend to her without a report from the police. She had to go to the police. She went to the police and then went back to the hospital, she was attended to and given medication. She went back to the police, and they told her they had to pick up her husband to hear his views or side of the story.” (Young married women, persona narration)

A healthcare worker also explained how fragmented services might further disadvantage survivors in the aftermath of violence.

“There is no one-stop centre, from [peri-urban area], this woman is badly beaten or raped, this woman comes to [our] health centre. You will find that this health person won’t attend to her unless she gets a police medical report. So, this survivor will go to police, get a medical report but why not just see her, document and in fact give her a referral to the police. So that she doesn’t have to explain much what they write, the police officer will just read that medical report. So, we lack information.” (Healthcare worker)

#### Resource limitations compounding existing challenges in seeking justice

Adding to confusing processes, participants also described how resource limitations further compounded barriers to post-violence support from police and legal services. Poor infrastructure and inconsistent information deterred participants from pursuing charges against perpetrators—whether these were their husbands, partners, or strangers. Critically, the lack of resources required to successfully pursue cases led to a negative feedback loop where survivors felt that justice was not possible, and not prioritized, for their experiences.

Limited resources for basic provisions—including transportation and fuel—meant that legal cases against perpetrators could stall, or never materialize at all. A police liaison provided context to this persistent challenge, justifying the delays and noting that police themselves often contributed to shortfalls:

Participant: “We only have one vehicle [for] the whole station. So you find someone brings a report on defilement or rape—how will they move without transport? So we request if they can manage transport or a cab to pick him up or they wait until the police vehicle is free. Because you cannot follow someone without that.”

Facilitator: “So if they can’t manage to find that cab money?”

Participant: “We contribute, us officers, from our pocket, we do assist if we have sometimes.” (Community member)

However, participants reiterated a sense of disempowerment that came from waiting. As one young woman explained:

“At the police station, if I go, I will explain [what happened] to them. The police will ask me what I want to do. . .the police will also answer you that, ‘find fuel, we go and arrest that person.’ And at this point, you do not have even a coin. So that also becomes stress[ful], because you need to find money to give the police officers and then you also need to go to the clinic. So that is also stress when you put everything together.” (Young married woman)

One survivor was asked to help locate the house of the person who had assaulted her:

“I felt like even if I do not forgive [the perpetrator], the police will not help. They will not have fuel, they will not help me look for the house, and I cannot go and look for a house that I do not know.” (Young GBV survivor)

Others reiterated similar situations in their personal narratives, and one survivor described how this delay affected her resolve to pursue a case:

“I think just the police, they made me feel lazy. It was in the night around 22h00, 23h00, and they told me to find transport for fuel. And we did not have [it], they need to be having fuel in their vehicles just there at the station, because sometimes the person who has beaten you is not even far, but if you have to look for money, you will cool down and start thinking about forgiving that person. At least the police should take fast action.” (Young GBV survivor)

Healthcare workers, too, echoed this sentiment in noting how systemic delays for survivors of sexual assault contributed to feelings of fatigue and disenchantment:

“One thing to bear in mind is [for] these sexual [assault] cases, the results take long to come out. . .maximum is 2 weeks and those are the challenges we have with sexual cases. Cause you find that at the end of the day, the survivor wants to give up, I think am tired of everything, because the results usually take long.” (Healthcare worker)

### Shifts in norms and practices

A final theme was identified around responses to inadequate support and service provision, highlighting normative shifts away from victim-blaming and revealing a more nuanced set of practices to navigate violence in intimate spaces. Notably, these accounts did not focus on survivors of non-partner violence, revealing how different available strategies relied on context.

Despite detailed discussion around stigma, secrecy, and limited family support for married women who experience violence, some participants described how women could protest against ongoing abuse through leveraging police services. One young woman described this as teaching one’s husband “a small lesson” about what is acceptable, thus exercising a form of agency:

“So, it’s better to teach him a lesson and take him to the police. . .you take a report so that they can teach him a small lesson. A man who loves you will worry about how he hurt you or what he did for you to act the way that you did.” (Young married woman)

Later in the conversation, participants in the young married women’s focus group returned to this topic:

Participant 1: “At the police, you will go and report. You will report that your husband has beaten you. They will ask why and then give you a medical report form to see a doctor. Then the doctor must put his signature and then you take the document to the police. Then you have the final say on whether to arrest him or not.”

Facilitator: “They ask so you can refuse if you want? They ask if you want him arrested or not?”

Participant 2: [*others talking at the same time*] “Some women will say just scold him, but don’t arrest him, he will listen to you.”

Participant 1: “Others just want to spend a night there.”

Participant 2: “Some just want him to be taught a lesson.”

(Young married women)

These contradictions echoed in another focus group conversation with a range of community members. Community members, including those who were religious leaders and traditional marriage counsellors, spoke to how their accepted approaches to marital counseling had changed, noting “those teachings are gone” and emphasizing the importance of disclosing violence and seeking help.

“We used to teach children that if your husband does this or that, don’t tell anyone, keep it to yourself. But these days we stopped with such traditional teachings. What we do these days is we tell them openly there is no secret if a man is beating you or abusing you in any way. . .Because next time it will be big, he can kill you.” (Community member)

“Times have changed, violence is too much such that it leads to death. A lot of people have died in marriages. If we as a church see the extent or damage that has been done to that family, in some instances we allow people to go separate ways. . .As a church we preach about forgiveness but if we see it doesn’t work, at some extent we have allowed people to go their separate ways in order to save a life.” (Community member)

These narratives positioned secrecy and shame alongside death, painting a stark image of what could happen within marriages where individuals hid violence. Certain perspectives retained a more paternalistic tone, emphasizing reconciliation and conflict resolution with an aim to keep the marriage together:

“At the end of the day we encourage reconciliation. Another thing, a woman can be beaten but not hurt, we sit them down and teach them how to live with each other. Don’t fight, involve elders to help you sort out your problems. . .So, we advise them on how to live, we talk to them. If it’s too much, an assault, we advise them to go to the police, they will counsel them. And the family proceeds with counsel from the police.” (Community member)

One healthcare worker specifically emphasized the importance of including traditional leadership in shifting harmful norms that underpin violence within families.

“Get rid of traditional norms, such as ‘a man’s unfaithfulness does not destroy his home.’ Especially this one, and the other one that, ‘I stay because of my children.’ Someone can be beaten with broken bones. . .with a lot of scars, but they say, ‘if I leave, who will take care of my children, who will give me money?’ So, we need to get rid of them. Yeah, then traditional counsellors should be involved in GBV meetings and trainings, right? Leaving no one behind, the traditional marriage counsellors and the headmen. . .if we engage them more at least they play a big role in changing these traditional norms.” (Healthcare worker)

## Discussion

Our findings highlight several key considerations about how adolescent girls and young women experiencing violence navigate both social norms and tangible services in peri-urban Lusaka. This study enabled us to probe both the deeper underlying norms and expectations surrounding how violence against girls and women is managed—silence, secrecy, and acceptance among them—as well as actual narratives on service provision. While our findings are tied to a specific context, they also carry implications for other similar settings, and echo the broader global imperative toward gender equality set out by the sustainable development goals.

Many adolescent girls and young women shared narratives that described a sense of being caught between worlds—between an older and newer generation, as well as between their childhood homes and an independent or a married life. In more “traditional” domestic and religious spaces (often the domains of families and elders), norms that are rooted in patriarchal, religious, and communal practices were described as disadvantaging survivors of violence. These included both inequitable gender norms as well as social norms and expectations, which appear across other qualitative work on gender norms and violence.^26^ Our participants indicated that social harmony was at times prioritized over the well-being of the individual survivor, which has been found in other diverse contexts, such as rural China.^27^ Survivors were expected to forgive or forget about violence for the sake of this communal harmony.

Related to this, we also found that norms were unevenly applied based on situations surrounding violence—for instance, whether women experienced violence within marriage versus violence outside of marriage. The GBV survivors who we engaged were recruited through one-stop centers, and were, by and large, adolescent girls and young women who had been assaulted by those other than their partners. There were structures in place to support them, and accessing this kind of support was socially accepted and even encouraged. Support-seeking following violence was more intermittent when this happened within marriages, and often started in more informal spaces, a finding that aligns with patterns in other global settings. A recent study of help-seeking behaviors for intimate partner violence during pregnancy, including data from 54 low- and middle-income countries, found that just over half of women sought help, and this happened mostly through informal channels.^28^ In our conversations, marital counseling was described as an accepted mode of help-seeking, yet our findings indicated that other external sources of support likely required more severe circumstances to be socially justifiable.

Concerning more tangible service provision, we found that young women’s difficult experiences accessing services reinforced negative views on support-seeking, and deepened mistrust in systems designed to protect women and girls. Even when survivors did seek support, they were sometimes marginalized or disregarded by the same system meant to serve them. These findings are echoed in previous work on support-seeking among survivors of intimate partner violence and GBV, including in sub-Saharan African settings^29^ and among younger survivors.^30^ This experience was made clear in the case of the survivor whose case updates were inappropriately shared with the perpetrator’s family, impeding the course of justice for this individual. Importantly, diverse experiences of discouragement, fragmentation, and duplication marred future interactions, or peers’ interactions, with these services in times of crisis. This erosion of trust in core post-violence support services was cast as disincentivizing girls and women from seeking services for violence—acting as a broader social cue about what was considered a priority.

However, there was also evidence that pointed to cracks in these negative norms, practices, and responses. For married women, drawing on existing resources to sanction their husbands, but not necessarily formally pressing charges, enabled them to signal their resistance to violence while staying within a socially acceptable space. In the community members’ conversation, several religious leaders and representatives put forward moderated perspectives, pushing back against what used to happen and indicating that they had had to undergo shifts in their own way of thinking and approaching violence. This kind of reframing, especially from individuals and institutions that perpetuate patriarchal norms and gender inequality, can be a critical step in norms change and violence outcomes.^31,32^ At the same time, there was a misalignment with what adolescent girls and young women voiced as viable options for support (barriers and discouragement from elders, and even peers, was a prominent theme) versus how certain community members and service providers described their own services as having evolved.

### Recommendations and key next steps

Taken together, these findings speak to a broader need for communication, and coordination, across partners and sectors to shift both harmful norms as well as ways of providing post-violence care. First, this relies on including a diversity of voices in these conversations: framing the continued need to have young women and men from diverse backgrounds, and with a range of life experiences, in these dialogs. Community conversations, the means through which we generated these key insights, proved to be an important mode of interaction, exchange, and learning; providing more community-based dialogs and public campaigns could produce both a better understanding among providers about the gaps and service blockages, as well as provide clarity for individuals (including but not limited to girls and women) about the steps to take in the aftermath of experiencing violence. In settings with limited resources, school- and community-based campaigns and community organization-led events may be more effective. These findings also reiterate the importance of including traditional leadership in addressing harmful norms that underpin violence. Traditional and religious leaders have the unique ability to influence their broader communities by drawing on their cultural authority and moral influence.^32^

Similarly, service providers, and those who design health and crisis response systems, need to be cognizant of how context can influence and constrain decision-making. For instance, our participants reiterated at multiple times how leaving a marriage is not necessarily a desirable option for many young women without broader support networks. Our previous publication from young women’s community conversations likened divorce to social death and pointed to ostracization and shame being a possibly greater source of emotional distress for women in certain situations.^6^ Supporting and working with couples may be one important approach within this reality, evidenced by promising approaches from diverse settings such as Nepal and Rwanda.^33,34^ Traditional marriage counsellors might be engaged in conversations about personal safety, harmful norms, promoting safe relationships, and navigating conflict in healthy ways, to help mitigate risk for women in these situations. The timing of marriage may also be important to integrate into these conversations—as maturity may bring better-quality couple interactions. To this end, targeting child, early, and forced marriages is a critical focus area to prevent and reduce violence and power imbalances. At the health system interface, training providers to be better sensitized to violence, especially among adolescent girls and young women, may add similar benefit.

Importantly, drawing out some of the contradictions in policy and practice is critical, in Zambia and elsewhere. Better infrastructure, resources, and service access could help support a more positive normative vision for how to address violence, from the top down. Training in trauma-informed approaches to care could enhance interactions and reduce secondary victimization across all violence-response systems that women are required to navigate—police, social services, and healthcare. Expanding training and resources to enable more nurses to conduct post sexual-assault examinations, for instance, may promote opportunities for both post-violence healthcare and justice. In practice, this priority clashes with existing, but poorly resourced, systems.^16^ Broader service improvements, through resource allocation and monitoring, could enhance responses to GBV. One-stop centers, for instance, have shown promising indications of effectiveness in Zambia and other low-resource settings, and are seen as a vital means of investing in GBV responses in the Global South.^35–37^ However, more research could support our understanding of the contexts in which they work best and the persisting gaps or challenges. These improvements could also help reframe norms in the process, across clinical, legal, and community-based institutions.

### Strengths and limitations

Our study built on a robust participatory approach to engage diverse groups of stakeholders. Young men, apart from one male peer supporter, were missing from our community conversations, and could have provided valuable insights. Additionally, our engagements were limited to one full week; while our project scope did not allow for repeat engagements with the same participants, these may have provided deeper insights over time.

## Conclusion

Our study’s insights, while context-specific, speak to broader considerations for shifting norms in the context of persistent experiences of violence in girlhood and early adulthood. For adolescent girls and young women transitioning to adulthood, identifying ways to better structure systems of support, and align them with healthier norms, can have lasting impacts on their well-being—and that of their partners, families, and communities.

## Supplemental Material

sj-docx-1-whe-10.1177_17455057261435814 – Supplemental material for Perspectives on post-violence support-seeking and service provision among adolescent girls and young women: Insights from community conversations in Lusaka, Zambia Supplemental material, sj-docx-1-whe-10.1177_17455057261435814 for Perspectives on post-violence support-seeking and service provision among adolescent girls and young women: Insights from community conversations in Lusaka, Zambia by Christina Laurenzi, Chuma Busakhwe, Chanda Mwamba, Chipo Mutambo, Eugene Mupakile and Elona Toska in Women's Health
