# Supplementary material for: Perspectives on post-violence support-seeking and service provision among adolescent girls and young women: Insights from community conversations in Lusaka, Zambia
Source: Womens Health (Lond). 2026 Apr 3;22:17455057261435814. doi: 10.1177/17455057261435814 (PMC13051140; doi:10.1177/17455057261435814)
Supplement: sj-docx-1-whe-10.1177_17455057261435814 – Supplemental material for Perspectives on post-violence support-seeking and service provision among adolescent girls and young women: Insights from community conversations in Lusaka, Zambia [file sj-docx-1-whe-10.1177_17455057261435814.docx]

**Supplemental File 1: Example session guides**

*Note: For any further use or borrowing of these activities, please cite the publication and acknowledge adaptation or borrowing of specific activities.*

**Session 1: Focus Group Interview Guide for AGYW GBV Survivors**

**Project Title:** Responding to violence including GBV among young women living with HIV in Zambia through peer supporter-clinic teams: the Screen & Support approach

**Ice Breaker:** To begin our discussion today, I want you to tell me some activities that you usually do in a day.  **Can you tell me more about what you do on a typical day?**

**Setting the context for the discussion**

**PART A. GBV among AGYW- Lived experiences**

GBV survivors Journey Mapping

Would you share with me your experience with GBV?

- To start with, let us reflect on our experiences of GBV and write our stories on these flip charts (give instructions and practical guidance).
- Re-group and discuss illustrations (PART A- circumstances at the time;, thoughts, feelings & concerns; PART B- information and influences; action taken; pain points and opportunity)

**Discussion**

1. What are the reasons for GBV among people your age?
   - 1. What are some of the main/common reasons for GBV?
2. What are the impacts that GBV has on young people your age in their day-to-day lives?
3. What are the types of GBV that are experienced by people your age in your community?
   - 1. What are the common types of GBV experienced by AGYW or people your age in your community? (Probe)
4. What are some of the factors/situations that may expose AGYW to GBV (risk)
5. Could you share with me if there are any specific groups of adolescents your age who are more likely to experience GBV? (Probe for people living with HIV, AGYW who were orphaned by HIV, adolescents with mental health problems, older adolescents, younger adolescents).
6. What do people in the community say about GBV?
7. How are AGYW your age who experience GBV treated by community members?
8. Is there anything else that you want to add about GBV among AGYW your age in the community?

**PART B (refer to personas): Gender-based violence services: mapping existing resources available and referral pathways for supporting GBV victims**

1. I would like to now discuss your opinions on services and programs that might be available for AGYW your age who experience GBV or feel at risk.
2. Did you seek assistance for the GBV?
   - 1. Probe: What/ who influenced your decision to seek help.
     2. How much do you think AGYW your age is aware of existing services or programs?
3. What would you do if you knew about a peer/ AGYW your age who might experience GBV?
4. How do communities respond to GBV (probe: what types of violence/situations likely to be reported/not reported)
5. What are some of the services or programs you are aware of that try to prevent AGYW your age from experiencing GBV**?**
6. What are some of the services or programs you are aware of that try to treat or help AGYW your age who have or are experiencing GBV?
7. Would you and other AGYW your age who experience GBV be willing to discuss their experiences with someone?
   - 1. What are some reasons that adolescents who experience GBV might not want to talk about it with other people?
     2. In what settings would AGYW be willing to discuss this?
     3. What types of individuals would AGYW be willing to discuss these experiences with?

**Understanding the GBV response in the Zambian context: service access facilitators and barriers to GBV care**

1. What are some of the challenges that AGYW your age who experience GBV might have in accessing programs or services to help them?
2. What are some things that might help AGYW access programs or services to help them?
3. Is there anything else that you want to add about GBV services and programs to care for AGYW in the community?

**Screening AGYW living with HIV for GBV risk and experiences through clinic teams**

I would now like to ask for your input on a potential program that could be used to screen and connect AGYW at risk or experiencing GBV to relevant care services. This program would screen AGYW who present at the health facility to get an indication about whether the AGYW is at risk or experiencing GBV. We would like to get your opinions on this.

I would like you to imagine an adolescent named Musimbi. Musimbi is 19 years old and is living with HIV. She receives care at [xxx] clinic. During a regular visit to [xxx] clinic to receive her medication and meet with a nurse, she is asked a number of questions about whether she feels at risk of or experiencing any form of GBV by the Peer Supporter.

OK, let’s talk a little bit together about this.

1. First, what are your general thoughts and feelings about this type of program? Probe how best can such a program be delivered at a health facility?
2. How do you think adolescents like Musimbi/yourself would feel about being asked about GBV during their regular visits to HIV care programs?
3. Can you talk about whether you think Musimbi would provide honest answers about her experience with GBV? Why or why not?
4. How do you think adolescents like Musimbi and yourselves would feel about getting referred to GBV services during their regular visits to HIV care programs?
5. What aspects of asking about GBV in these settings do you think adolescents like Musimbi/yourself would like or not like?
6. Can you tell us if you think that having a GBV program like this within the HIV care visit would make an adolescent like Musimbi/yourself more or less likely to attend the HIV care visit? What are the reasons that you think this?
7. What are some things that might make adolescents like Musimbi/yourself more comfortable with this type of program?
8. Which health cadre do you think is best suited to conduct the GBV screening? Why. Probe Peer Supporters?
9. What are challenges, barriers, or obstacles to this type of program?

**….**

**Session 3: Focus Group Interview Guide for Health Service Providers including Peer Supporters**

**Ice Breaker:** To begin our discussion today, I want you to tell me briefly about your work at the health facility.

**Setting the context for the discussion**

**GBV among AGYW- Community context**

I would now like to talk about GBV among AGYW in your communities and whom you see in the facility.

1. What is your perception of GBV?
2. What do people in the community say about GBV?
3. What are the types of GBV that are experienced by AGYW in your community?
   - 1. What are the common types of GBV experienced by AGYW in your community? (Probe)
4. What are some of the factors/situations that may expose AGYW to GBV (risk)
   - 1. Can you describe a typical scenario when AGYW may be subjected to GBV (type of gender based violence/scenario)?
5. Could you share with me if there are any specific groups of adolescents who are more likely to experience GBV? (probe for people living with HIV, AGYW who were orphaned by HIV, adolescents with mental health problems, older adolescents, younger adolescents).
6. What are the reasons for GBV among AGYW?
   - 1. What are some of the main/common reasons for GBV?
7. What are some of the effects/impacts that are caused by GBV among AGYW on their daily lives?
8. How are AGYW who experience GBV treated by community members?
9. Is there anything else that you want to add about GBV among AGYW in the community?

**Gender-based violence services: mapping existing resources available and referral pathways for supporting GBV victims**

1. I would like to now discuss your opinions on services and programs that might be available for AGYW who experience GBV.
2. What are some of the services or programs you are aware of that try to prevent AGYW from experiencing GBV**?**
3. What are some of the services or programs you are aware of that try to treat or help AGYW who have or are experiencing GBV?
4. Probe: How much do you think AGYW are aware of existing services or programs?
5. How do communities respond to GBV (probe: what types of violence/situations likely to be reported/not reported)
6. Could you tell us if you think AGYW who experience GBV would be willing to discuss their experiences with someone?
   - 1. What are some reasons that adolescents who experience GBV might not want to talk about it with other people?
     2. In what settings would AGYW be willing to discuss this?
     3. What types of individuals would AGYW be willing to discuss these experiences with?

**Understanding the GBV response in the Zambian context: service access facilitators and barriers to GBV care**

***Free listing: break into groups by health care worker cadre type. Regroup for discussion.***

1. What are some of the challenges that AGYW who experience GBV might have in accessing programs or services to help them?
2. What are some things that might help AGYW access programs or services to help them?
3. Is there anything else that you want to add about GBV services and programs to care for AGYW in the community?

**Screening AGYW living with HIV for GBV risk and experiences through clinic teams**

I would now like to ask for your input on a potential program that could be used to screen and connect AGYW at risk or experiencing GBV to relevant care services. This program would screen AGYW who present at the health facility to get an indication about whether the AGYW is at risk or experiencing GBV. We would like to get your opinions on this.

I would like you to imagine an adolescent named Musimbi. Musimbi is 19 years old and is living with HIV. She receives care at [xxx] clinic. During a regular visit to [xxx] clinic to receive her medication and meet with a nurse, she is asked a number of questions about whether she feels at risk of or experiencing any form of GBV by the Peer Supporter.

OK, let’s talk a little bit together about this.

1. First, what are your general thoughts and feelings about this type of program? Probe how best can such a program be delivered at a health facility?
2. Which health cadre do you think would be best suited to screen for GBV among AGYW? Why/why not
3. What are your thoughts about considerations to use Peer Supporters to conduct this screening? What are the barriers or challenges to peers in this role
4. What would be the training points to equip health workers with skills to screen for GBV
5. How do you think adolescents like Musimbi would feel about being asked about GBV during their regular visits to HIV care programs?
6. Can you talk about whether you think Musimbi would provide honest answers about her experience with GBV? Why or why not?
7. How best do you think adolescents like Musimbi should be referred to GBV services after screening and found at risk or experiencing GBV? Probe steps, systems, resources
8. Can you tell us if you think that having a GBV program like this within the HIV care visit would make an adolescent like Musimbi more or less likely to attend the HIV care visit? What are the reasons that you think this?
9. What are challenges, barriers, or obstacles to this type of program?

**Show charts with screening items**

1. What kinds of screening items might be best positioned in these settings to pick up violence?
2. What kinds of screening items asking about GBV in these settings do you think adolescents may find most appropriate / acceptable to make them more comfortable with the program?
3. What kinds of screening items asking about GBV in these settings do you think adolescents like Musimbi would not like?

**CLOSING**

1. Is there anything else that anyone would like to say about any of the topics we have talked about today?
2. Thank you all very much for your time and for your input; your opinions are very helpful.
